# Better Myocardial Function in Aortic Stenosis with Low Left Ventricular Mass: A Mechanism of Protection against Heart Failure Regardless of Stenosis Severity?

**DOI:** 10.3390/jcm8111836

**Published:** 2019-11-01

**Authors:** Bernadeta Chyrchel, Klaudiusz Bolt, Dorota Długosz, Anna Urbańska, Małgorzata Nowak-Kępczyk, Joanna Bałata, Agnieszka Rożanowska, Ewa Czestkowska, Olga Kruszelnicka, Andrzej Surdacki

**Affiliations:** 1 Second Department of Cardiology, Jagiellonian University Medical College, 31-501 Cracow, Poland; chyrchelb@gmail.com; 2 Students’ Scientific Group at the Second Department of Cardiology, Jagiellonian University Medical College, 31-501 Cracow, Poland; k.bolt@student.uj.edu.pl (K.B.); doris.dlugosz@gmail.com (D.D.); anna.uurbanska@gmail.com (A.U.); aga244@poczta.onet.eu (A.R.); ewaczestkowska@gmail.com (E.C.); 3 Institute of Mathematics and Computer Science, The John Paul II Catholic University of Lublin, 20-708 Lublin, Poland; gosianmk@wp.pl; 4 Center of Postgraduate Education for Nurses and Midwives in Rzeszów—Tarnobrzeg Branch, 39-400 Tarnobrzeg, Poland; asia.balata@gmail.com; 5 Department of Coronary Artery Disease and Heart Failure, Jagiellonian University Medical College, 31-202 Cracow, Poland; olga.kruszelnicka@gmail.com

**Keywords:** aortic stenosis, left ventricular hypertrophy, myocardial contractility, hemodynamic load

## Abstract

About one-tenth to one-third of patients with severe aortic stenosis (AS) do not develop left ventricular hypertrophy (LVH). Intriguingly, the absence of LVH despite severe AS is associated with lower prevalence of heart failure (HF), which challenges the classical notion of LVH as a beneficial compensatory response. Notably, the few studies that have attempted to characterize AS subjects with inadequately low left ventricular (LV) mass relative to LV afterload (i-lowLVM) described better prognosis and enhanced LV performance in AS associated with i-lowLVM, but those reports were limited to severe AS. Our aim was to compare myocardial function between moderate and severe AS with i-lowLVM. We retrospectively analyzed in-hospital records of 225 clinically stable nondiabetic patients with isolated moderate or severe degenerative AS in sinus rhythm, free of coexistent diseases. Subjects with i-lowLVM were compared to those with appropriate or excessive LVM (a/e-LVM), defined on the basis of the ratio of a measured LVM to the LVM predicted from an individual hemodynamic load. Patients with i-lowLVM and a/e-LVM did not differ in aortic valve area, LV end-diastolic diameter (LVd, a measure of LV preload), and circumferential end-systolic LV wall stress (cESS), an estimate of LV afterload. Compared to a/e-LVM, patients with i-lowLVM had increased LV ejection fraction (EF) and especially higher LV midwall fractional shortening (a better index of LV myocardial function than EF in concentric LV geometry) (*p* < 0.001–0.01), in both moderate and severe AS. LVd and cESS were similar in the four subgroups of the study subjects, i.e., moderate AS with i-lowLVM, moderate AS with a/e-LVM, severe AS with i-lowLVM, and severe AS with a/e-LVM (*p* > 0.6). Among patients with i-lowLVM, LVM did not differ significantly between moderate and severe AS (*p* > 0.4), while in those with a/e-LVM, LVM was increased in severe versus moderate AS (*p* < 0.001). In conclusion, the association of the low-LVM phenotype with better myocardial contractility may already develop in moderate AS. Additionally, cESS appears to be a controlled variable, which is kept constant over AS progression irrespective of LVM category, but even when controlled (by increasing LVM), is not able to prevent deterioration of LV function. Whether improved myocardial performance contributes to favorable prognosis and the preventive effect against HF in AS without LVH, remains to be studied.

## 1. Introduction

About one-tenth to one-third of patients with severe aortic stenosis (AS) do not develop left ventricular hypertrophy (LVH) [1,2,3,4]. Intriguingly, the absence of LVH despite severe AS was associated with better left ventricular (LV) systolic performance [1,2] and threefold lower prevalence of heart failure (HF) [2], which challenges the classical notion of LVH as an adaptive response to pressure overload, aimed at the preservation of LV systolic performance via the normalization of LV wall stress. Moreover, prognosis was neither worsened [3] or even better [2,4] in subjects with severe AS without LVH, including symptomatic or asymptomatic AS subjects and those undergoing surgical aortic valve replacement. 

Of note, only a few studies have attempted to characterize patients with inadequately low LV mass (i-lowLVM), out of proportion to LV afterload. In hypertensives with a measured LVM below the 2.5th percentile of the distribution of the LVMpredicted from individual hemodynamic parameters and compared with a reference population, De Simone et al. [5] described enhanced afterload-corrected LV myocardial function. Importantly, in that study [5], the rate of future cardiovascular (CV) events was the same as in those with appropriate LVM. With regard to severe AS, Cioffi et al. [4] reported a reduced risk of adverse CV outcome in subjects in the lowest tertile of the observed-to-predicted LVM ratio, and we have recently observed an improved LV myocardial and chamber function despite comparable afterload in subjects with severe AS and i-lowLVM, defined as a LVM below the 23th percentile of the predicted LVM [6]. 

To the best of our knowledge, characteristics of subjects with i-lowLVM and moderate AS, an antecedent of severe AS, have not been reported. Recently, Ito et al. [7] found that afterload-corrected LV systolic function was depressed in 17% of patients with moderate AS and ejection fraction (EF) ≥60%, and further decreased during the progression into severe AS, in contrast to the remaining AS subjects with preserved EF in whom stress-corrected LV performance did not change over time. This finding can reflect a distinct type of LV mechanics in a subset of AS subjects, irrespective of disease severity. Since left ventricular mass (LVM) inadequacy probably also represents a different pattern of LV response to pressure overload [2,6], which might appear already at early stages of AS, our aim was to compare myocardial function between moderate and severe AS with i-lowLVM.

## 2. Materials and Methods

### 2.1. Patients

We performed a retrospective analysis of medical records of patients electively hospitalized in our department with a confirmed discharge diagnosis of moderate or severe degenerative AS, with preserved sinus rhythm. AS severity was determined by means of echocardiography done by an experienced sonographer, following the clinical practice guidelines [8,9,10], on the basis of aortic valve area (AVA) (moderate AS: AVA = 1.0–1.5 cm^2^; severe AS: AVA < 1.0 cm^2^). 

As previously described [6,11], we had excluded clinically unstable subjects and those with coexistent diseases: more than mild aortic incompetence or disease of another valve, relevant coronary artery disease (CAD) (i.e., a history of acute coronary syndrome, coronary angioplasty or by-pass surgery, or significant narrowings of major epicardial coronary arteries on angiography), EF below 50%, any clinically relevant noncardiovascular comorbidity, or with significant abnormalities in routine blood and urine analysis. 

Since we have recently described impaired LV systolic performance in AS with concomitant type 2 diabetes [11], AS subjects with any endocrine disorders, including diabetes, were excluded from the analysis. As an association between lower estimated glomerular filtration rate (GFR), decreased LV systolic performance, and inappropriately high LVM relative to LV afterload was reported in our earlier work [11], patients with chronic kidney disease had also been excluded.

In order to avoid analyzing mainly the same patients as in previous our reports [6,11,12], the present study was based on medical records of predominantly newly recruited nondiabetic AS subjects, using the above described inclusion and exclusion criteria. The bioethical committee of our university renewed the approval of our retrospective study, including also a waiver of the requirement for patient’s informed consent (renewal issued on 31 Jan. 2019; No. 122.6120.228.2016). 

### 2.2. Analysis of Medical Records

LVM was calculated from in-hospital echocardiography by the modified Devereux formula [13]. LVM adequacy was estimated as earlier described in detail [4,5,6,14]. In brief, we first computed the predicted LVM by the previously validated formula from height, gender, stroke volume, maximal aortic pressure gradient, and mean systolic blood pressure, averaging all blood pressure measurements performed during the index hospitalization [4,5,6]. Then, the ratio of LVM_measured_ to LVM_predicted_ was computed for each AS subject and LVM inadequacy was defined based on the distribution of this ratio in the study patients [4,5,6]. Accordingly, i-lowLVM corresponded to the first quintile of the LVM_measured_-to-LVM_predicted_ ratio (i.e., LVM_relative_), while the remaining AS patients included those with both appropriate and inappropriately high LVM (i.e., excessive LVM), who were analyzed jointly.

From routine in-hospital echocardiography, we estimated LV systolic performance both at the endocardial and myocardial level, reflecting LV chamber and myocardial function, respectively. LV chamber function was represented by EF calculated by the biplane Simpson’s method [13], whereas LV myocardial function was quantified as LV midwall fractional shortening (mwFS). In accordance with a cylindrical LV model, mwFS was assessed, assuming a constant volume of the LV inner shell (i.e., between the endocardium and LV midwall) during systole and diastole [5,6,15,16], thereby providing a better measure of LV performance than conventional EF in patients with concentric LVH [17], frequently observed in AS. Additionally, in order to estimate LV afterload, we calculated circumferential end-systolic LV wall stress at the midwall level (cESS) from mean systolic blood pressure, maximal aortic pressure gradient, and LV end-systolic internal diameter and posterior wall thickness, as previously proposed [16]. The formula for cESS—developed in 1972 by Gaasch et al. [18] and modified later [19,20]—was applied in experimental studies [19,20] and validated in patients with aortic valve disease [21]. Also, LV end-diastolic internal diameter was recorded as an approximate index of LV preload. 

### 2.3. Statistical Analysis

Data are shown as mean and standard deviation, or numbers and percentages. Patients’ characteristics were compared between the following 4 subgroups with equal proportions of women and men: moderate AS with i-lowLVM (*n* = 23), moderate AS with appropriate or excessive LVM (a/e-LVM) (*n* = 92), severe AS with i-lowLVM (*n* = 22), and severe AS with a/e-LVM (*n* = 88). Intergroup differences in continuous variables were assessed by 2-way ANOVA, followed by the post hoc Scheffe’s test. Dichotomous data were compared by the Chi-square test. 

In addition, Pearson’s correlation coefficients (r) were calculated to assess the relationship between mwFS, cESS, and LVM_relative_, both for all study subjects and only those with i-lowLVM. Then, multivariate linear regression was used to estimate independent determinants of mwFS with the following covariates: cESS, AVA, LV end-diastolic diameter, age, and the ratio of a measured LVM and the LVM predicted from an individual hemodynamic load (LVM_relative_). Finally, to illustrate effects of cESS and LVM_relative_ on mwFS, mwFS was compared by Student’s *t*-test between patients with a below-median cESS (<201 kdynes/cm^2^) and above-median cESS (>201 kdynes/cm^2^), categorized according to the distribution of LVM_relative_: i-lowLVM (LVM_relative_ < 20th percentile), LVM_relative_ between 20th and 80th percentile, and LVM_relative_ > 20th percentile. Trend effects of increasing LVM categories on mwFS were assessed by Spearman’s rank-order correlation coefficient (rho). 

A *p*-value below 0.05 was assumed significant.

## 3. Results

Patients with moderate AS and i-lowLVM did not differ from their counterparts with moderate AS and a/e-LVM in terms of clinical (Table 1) and echocardiographic (Table 2) characteristics, except for higher EF (65 ± 7 vs. 58 ± 8%, *p* < 0.01) and mwFS (18.9 ± 2.6 vs. 14.0 ± 3.3%, *p* < 0.001) (Table 2). 

Likewise, EF and mwFS were increased in subjects with severe AS and i-lowLVM in comparison with those with severe AS and a/e-LVM (EF: 65 ± 9 vs. 56 ± 7%, *p* < 0.001; mwFS: 15.7 ± 3.1 vs. 13.0 ± 2.5, *p* < 0.01) (Table 2), while the remaining characteristics were similar among the respective subgroups (Table 1 and Table 2). In particular, cESS was almost identical regardless of LVM adequacy and stenosis severity (moderate AS with i-lowLVM: 211 ± 56 kdynes/cm^2^ hPa; moderate AS with a/e-LVM: 192 ± 83 kdynes/cm^2^; severe AS with i-lowLVM: 217 ± 67 kdynes/cm^2^; severe AS with a/e-LVM: 207 ± 81 kdynes/cm^2^, *p* > 0.6) (Table 2). 

Among patients with i-lowLVM, LVM did not differ significantly between moderate and severe AS (158 ± 42 g vs. 192 ± 58 g, respectively, *p* > 0.4), while in those with a/e-LVM, LVM was increased in severe versus moderate AS (299 ± 87 vs. 211 ± 74 g, *p* < 0.001) (Table 2).

In all study patients, mwFS correlated negatively to cESS (r = −0.44, *p* < 0.001) and LVM_relative_ (r = −0.46, *p* < 0.001), which was also found in patients with i-low LVM (mwFS vs. cESS: r = −0.53, *p* < 0.001; mwFS vs. LVM_relative_: r = −0.52, *p* < 0.001). cESS was unrelated to LVM_relative_ both in the study group as a whole (r = −0.06, *p* > 0.3) and in subjects with i-lowLVM (r = −0.08, *p* > 0.6). 

Upon multivariate analysis, lower mwFS was independently related to higher cESS (mean standardized regression coefficient [β] ± SEM: −0.50 ± 0.07, *p* < 0.001) and increased LVM_relative_ (β = −0.44 ± 0.07, *p* < 0.001). These effects were reflected by reduced mwFS in patients with an above-median vs. below-median cESS (*p* < 0.001 or *p* < 0.05), and gradual decreases of mwFS with increasing LVM categories at both a below-median cESS (rho = −0.46, *p* < 0.001) and an above-median cESS (rho = −0.54, *p* < 0.001) (Table 3). 

## 4. Discussion

Our principal finding was that LV systolic performance was better in patients with a low LVM disproportionate to LV afterload. This was already the case in patients with moderate AS, at a comparable cESS as in severe AS. This observation is in agreement with an early study by De Simone et al. [5], who described a preserved afterload-corrected mwFS in hypertensives with i-lowLVM, and supplements our previous report [6] of higher mwFS at similar cESS in severe AS and i-lowLVM in comparison to severe AS and a/e-LVM. 

To the best of our knowledge, our report is the first to describe the association of i-lowLVM with enhanced LV contractility in moderate AS. Nevertheless, our findings appear compatible with better LV systolic performance in patients with mild or moderate AS and absent LVH participating in the SEAS trial [22]. Although LVM adequacy was not estimated in that study, it does not seem implausible to assume that the majority of those AS patients without LVH also had i-lowLVM. In that report [22] cESS was similar irrespective of LVH presence, which is analogous to our observation of comparable cESS regardless of LVM adequacy relative to LV afterload. 

The fact that cESS was similar regardless of stenosis severity, LVM category, or LVM_relative_ as a continuous parameter, points to cESS as a controlled variable, which is kept constant over AS progression. The negative mwFS–cESS relationship, maintained in multivariate analysis, is likely to reflect the fundamental inverse stress-shortening relation, demonstrated in hypertension [16,17] and AS [14]. Notably, in addition to higher cESS, the ratio of a measured LVM to the LVM predicted from an individual hemodynamic load (LVM_relative_) was the second independent predictor of decreased mwFS, which is consistent with the study by Mureddu et al. [23] on AS and hypertension. The gradual decrease in mwFS with increasing LVM categories, especially pronounced in patients in the top quintile of the ratio, may correspond to the gradual transition from adaptive into maladaptive (i.e., excessive) LVH. Thus, the association of inappropriately high LVM with LV systolic dysfunction could be perceived as a partially inefficient compensatory mechanism aimed at the preservation of LV pump function through lowering cESS [14]. As proposed by Cioffi et al. [14], excessive LVH would develop in AS when the LVH-induced changes in LV geometry are no longer able to compensate for impaired intrinsic myocardial contractility despite low cESS, in analogy to earlier observations in hypertensive subjects with low levels of afterload [24]. 

In the present study, i-lowLVM was accompanied by higher mwFS, whereas a/e-LVM was accompanied by lower mwFS, regardless of AS severity, while no differences were found in cESS that was inversely correlated to mwWS. Thus, the mere presence of hypertrophy was associated with a decreased mwFS, especially in patients with increased cESS. Therefore, patients with i-lowLVM appear to represent a different mode of cardiac adaptation to pressure overload, which appears already in less than severe AS. This concept has been based on a hypothesis put forward almost 15 years ago by Kupari et al. [2], who observed postoperative LVM regression and improvement of EF over three months after valve replacementonly in AS subjects with preoperative LVH, in contrast to their counterparts without LVH despite critically severe AS, in whom higher preoperative EF and threefold lower prevalence of HF were found. However, Kupari et al. [2], Cioffi et al. [4], and Gerdts et al. [22]—who demonstrated beneficial prognostic effects of absent or inadequate LVH in AS—did not analyze a possible role of cESS for their findings. In our hands, mean cESS was similar among all study subgroups, which could suggest that persistently enhanced LV systolic function without concomitant afterload excess may contribute to improved outcomes in AS subjects with low LVM [1,2,3,4,22]. 

Admittedly, any conclusions are strongly limited by the fact that our cross-sectional analysis was based on only one echocardiographic examination. Nevertheless, in a recent longitudinal study by Ito et al. [7], cESS remained in the normal range in all 290 patients with moderate AS and EF ≥ 60%, and even decreased (by an average of 6%) during the next three years when the subjects progressed from moderate into severe AS. In addition, the prevalence of impaired LV contractility (represented by mwFS plotted against cESS) only slightly increased from 17% into 24% over three years in those with preserved EF [7]. In contrast, time-dependent increases in cESS were found exclusively in 155 subjects with EF < 60%, especially with coexistent depressed LV contractility [7]. Accordingly, in line with the previously mentioned earlier observations in AS [14,23], mwFS depression despite normal EF appears to result not from afterload mismatch, but from a discrete reduction of LV contractility, which already exists in less than severe valve disease in a subset of AS patients [7]. On the opposite side of the continuum of LV systolic performance, enhanced LV contractility might also be present in a subgroup of AS patients irrespective of stenosis severity, especially those with low LVM, although Ito et al. [7] did not take into account the type of LVH, i.e., adaptive or maladaptive.

This pattern of LV response to pressure overload probably accounts for the not worsened or even improved prognosis in AS patients with i-lowLVM [4] or absent LVH [1,2,3,22] associated with enhanced LV systolic function [1,2,6,22], observed also in AS subjects with i-lowLVM in the present study. Hence, potential drawbacks of LVM inadequacy are likely to be offset by possible benefits of a better LV systolic performance, as well as the protection against excessive LVH. Excessive LVH, associated with adverse CV outcome via a variety of hemodynamic and metabolic mechanisms [4,5,25,26,27,28,29,30], develops already in 16%–26% of patients with mild-to-moderate AS [14,23] and its frequency rises over AS progression [4,23]. Nevertheless, in order to clarify the predictive ability of LVH inadequacy in AS patients, large-scale prospective studies are warranted with simultaneous complex assessment of LV performance and loading conditions. 

### Study Limitations

First, our study was limited by a retrospective cross-sectional design; longitudinal observations of changes in LVM and LV performance over time would be more appropriate to investigate the relationship between LVM adequacy and LV function during AS progression. Moreover, duration to the development of severe AS is likely to affect both myocardial function and the magnitude of LVH. However, time from AS diagnosis and past echocardiographic records were available only in a minority of our AS patients. Furthermore, patients with moderate AS and i-lowLVM might progress into severe AS with a/e-LVM, which also limits the interpretation of our cross-sectional analysis due to the missing information on time-dependent changes of LVM and LV mechanics. Second, from available medical records, we were able to derive only mwFS as a better estimate of LV function than EF, while novel echocardiographic techniques, i.e., strain analysis [31], might provide a better insight into LV contractility. Third, we analyzed a heterogeneous group of consecutive AS subjects. Nevertheless, a wide set of exclusion criteria was aimed at limiting potential effects of coexistent diseases, and the proportion of women and men was equal in all four subgroups to eliminate potential gender-specific effects. On the other hand, we can not entirely exclude a bias resulting from the use of angiotensin-converting enzyme inhibitors and angiotensin antagonists, known to decrease the magnitude of LVH in both mild-to-moderate [32] and severe AS [33], nonetheless, the percentage of patients using these drugs was comparable across the study subgroups. 

## 5. Conclusions

The association of the low-LVM phenotype with better myocardial contractility may already develop in moderate AS. Additionally, cESS appears to be a controlled variable, which is kept constant over AS progression, but even when controlled (by increasing LVM), is not able to prevent deterioration of LV function. Whether improved myocardial performance contributes to favorable prognosis and the preventive effect against HF in AS without LVH, remains to be studied.

## Figures and Tables

**Table 1:** Comparison of clinical characteristics between aortic stenosis (AS) subjects with inadequately low mass relative to LV afterload i-lowLVM and appropriate or excessive LVM (a/e-LVM) stratified by AS severity.

Characteristic	Moderate AS		i-lowLVM vs. a/e-LVM *p*-Value	Severe AS		i-lowLVM vs. a/e-LVM *p*-Value
	i-lowLVM *n* = 23	a/e-LVM *n* = 92		i-lowLVM *n* = 22	a/e-LVM *n* = 88	
Age, years	69 ± 7	68 ± 8	NS	70 ± 8	70 ± 7	NS
Women/men, *n*	12/11	46/46	NS	11/11	44/44	NS
BMI, kg/m^2^	27 ± 4	28 ± 4	NS	27 ± 3	28 ± 3	NS
GFR, mL/min/1.73 m^2^	76 ± 13	77 ± 13	NS	77 ± 14	78 ± 13	NS
Hypertension, *n* (%)	19 (83%)	74 (80%)	NS	18 (82%)	71 (81)	NS
Mean BP, mmHg	92 ± 11	92 ± 9	NS	92 ± 10	91 ± 10	NS
Medication, *n* (%)						
ACEI or ARB	12 (52%)	43 (47%)	NS	8 (36%)	29 (33%)	NS
Beta blockers	12 (52%)	51 (55%)	NS	13 (59%)	47 (53%)	NS
Diuretics	9 (39%)	40 (43%)	NS	11 (50%)	38 (43%)	NS
CCB	8 (35%)	35 (38%)	NS	9 (41%)	34 (39%)	NS

Data are presented as mean ± standard deviation or numbers (percentages). ACEI: angiotensin-converting enzyme inhibitor; ARB: angiotensin receptor blocker; BMI: body mass index; BP: blood pressure; CCB: calcium channel blockers; GFR: estimated glomerular filtration rate by the CKD-EPI formula; LV: left ventricular; LVM: left ventricular mass.

**Table 2:** Comparison of hemodynamic characteristics between patients with i-lowLVM and a/e-LVM stratified by AS severity.

Characteristic	Moderate AS		i-lowLVM vs. a/e-LVM *p*-Value	Severe AS		i-lowLVM vs. a/e-LVM *p*-Value
	i-lowLVM *n* = 23	a/e-LVM *n* = 92		i-lowLVM *n* = 22	a/e-LVM *n* = 88	
AVA, cm^2^	1.2 ± 0.15	1.25 ± 0.2	NS	0.85 ± 0.2 †	0.8 ± 0.2 *	NS
PG_mean_, mmHg	29 ± 7	30 ± 8	1.2 ± 0.15	55 ± 14 †	57 ± 16 *	NS
LVd, mm	51 ± 5	50 ± 7	NS	48 ± 7	50 ± 8	NS
cESS, kdynes/cm^2^	211 ± 56 211 ± 56	192 ± 83	NS NS	217 ± 67	207± 81	NS
EF, %	65 ± 7	58 ± 8	<0.01	65 ± 9	56 ± 7	<0.001
mwFS, %	18.9 ± 2.6	14.0 ± 3.3	<0.001	15.7 ± 3.1	13.0 ± 2.5	<0.01
LVM, g	158 ± 42	211 ± 74	<0.01	192 ± 58	299 ± 87 *	<0.001
LVM_relative_	0.7 ± 0.2	1.3 ± 0.3	<0.001	1.0 ± 0.2	1.6 ± 0.3 *	<0001

* *p* < 0.001 vs. moderate AS and a/e-LVM; † *p* < 0.001 vs. moderate AS and i-lowLVM. Data are presented as mean ± standard deviation. Significant post hoc *p*-values are marked as bold. a/e-LVM: LVM_relative_ ≥ 20th percentile; AVA: aortic valve area; cESS: circumferential end-systolic LV wall stress; EF: ejection fraction; i-lowLVM: LVM_relative_ < 20th percentile; LV: left ventricular; LVd: LV end-diastolic internal dimension; LVM: LV mass; LVM_relative_: LVM_measured_-to-LVM_predicted_ ratio; mwFS: LV midwall fractional shortening; PG_mean_: mean aortic pressure gradient.

**Table 3:** LV midwall fractional shortening (mwFS) in relation to circumferential end-systolic stress (cESS) according to the categorized ratio of a measured LVM and the LVM predicted from hemodynamic load.

Stratification According to cESS	mwFS (%) According to the Distribution of the LVM_measured_-to-LVM_predicted_ ratio (LVM_relative_)			mwFS vs. LVM_relative_ *p*-Value for Trend
	Percentiles of LVM_relative_			
	<20th (i-lowLVM) *n* = 45	20th–80th *n* = 135	>80th *n* = 45	
Below-median cESS *n* = 112	18.6 ± 2.3	15.7 ± 2.4	13.9 ± 3.3	<0.001
Above-median cESS *n* = 113	16.6 ± 2.9	12.3 ± 2.9	10.5 ± 2.5	<0.001
*p*-value Below-median vs. above-median cESS	<0.05	<0.001	<0.001	

## References

[1] Seiler C., Jenni R. (1996). Severe aortic stenosis without left ventricular hypertrophy: Prevalence, predictors, and short-term follow up after aortic valve replacement. Heart.

[2] Kupari M., Turto H., Lommi J. (2005). Left ventricular hypertrophy in aortic valve stenosis: Preventive or promotive of systolic dysfunction and heart failure?. Eur. Heart J..

[3] Barasch E., Kahn J., Petillo F., Pollack S., Rhee P.D., Reichek N. (2014). Absence of left ventricular hypertrophy in severe isolated aortic stenosis and preserved left ventricular systolic function. J. Heart Valve Dis..

[4] Cioffi G., Faggiano P., Vizzardi E., Tarantini L., Cramariuc D., Gerdts E., de Simone G. (2011). Prognostic effect of inappropriately high left ventricular mass in asymptomatic severe aortic stenosis. Heart.

[5] De Simone G., Palmieri V., Koren M.J., Mensah G.A., Roman M.J., Devereux R.B. (2001). Prognostic implications of the compensatory nature of left ventricular mass in arterial hypertension. J. Hypertens..

[6] Chyrchel B., Długosz D., Bolt K., Kruszelnicka O., Dziewierz A., Świerszcz J., Wieczorek-Surdacka E., Hryniewiecki T., Surdacki A. (2018). Association of inadequately low left ventricular mass with enhanced myocardial contractility in severe degenerative aortic stenosis. J. Clin. Med..

[7] Ito S., Pislaru C., Miranda W.R., Nkomo V.T., Connolly H.M., Pislaru S.V., Pellikka P.A., Lewis B.R., Carabello B.A., Oh J.K. (2019). Left ventricular contractility and wall stress in patients with aortic stenosis with preserved or reduced ejection fraction. JACC Cardiovasc. Imaging.

[8] Vahanian A., Alfieri O., Andreotti F., Antunes M.J., Barón-Esquivias G., Baumgartner H., Borger M.A., Carrel T.P., De Bonis M., Evangelista A. (2012). Guidelines on the management of valvular heart disease (version 2012). Eur. Heart J..

[9] Baumgartner H., Falk V., Bax J.J., Bonis M., Hamm C., Holm P.J., Iung B., Lancellotti P., Lansac E., Munoz D.R. (2018). 2017 ESC/EACTS Guidelines for the management of valvular heart disease. Kardiol. Pol..

[10] Baumgartner H., Hung J., Bermejo J., Chambers J.B., Edvardsen T., Goldstein S., Lancellotti P., LeFevre M., Miller F., Otto C.M. (2017). Recommendations on the echocardiographic assessment of aortic valve stenosis: A focused update from the European association of cardiovascular imaging and the American society of echocardiography. J. Am. Soc. Echocardiogr..

[11] Czestkowska E., Rożanowska A., Długosz D., Bolt K., Świerszcz J., Kruszelnicka O., Chyrchel B., Surdacki A. (2019). Depressed systemic arterial compliance and impaired left ventricular midwall performance in aortic stenosis with concomitant type 2 diabetes: A retrospective cross-sectional study. Cardiovasc. Diabetol..

[12] Długosz D., Bolt K., Sam W.S., Nawara T., Kruszelnicka O., Chyrchel B., Surdacki A. (2018). Excessive left ventricular hypertrophy in moderate degenerative aortic stenosis: An ineffective compensatory mechanism triggered by primary myocardial dysfunction and enhanced by concomitant mild renal impairment?. Kardiol. Pol..

[13] Lang R.M., Badano L.P., Mor-Avi V., Afilalo J., Armstrong A., Ernande L., Flachskampf F.A., Foster E., Goldstein S.A., Kuznetsova T. (2015). Recommendations for cardiac chamber quantification by echocardiography in adults: An update from the American Society of Echocardiography and the European Association of Cardiovascular Imaging. Eur. Heart J. Cardiovasc. Imaging.

[14] Cioffi G., de Simone G., Cramariuc D., Mureddu G.F., Gerdts E. (2012). Inappropriately high left-ventricular mass in asymptomatic mild-moderate aortic stenosis. J. Hypertens..

[15] Shimizu G., Hirota Y., Kita Y., Kawamura K., Saito T., Gaasch W.H. (1991). Left ventricular midwall mechanics in systemic arterial hypertension. Myocardial function is depressed in pressure-overload hypertrophy. Circulation.

[16] De Simone G., Devereux R.B., Roman M.J., Ganau A., Saba P.S., Alderman M.H., Laragh J.H. (1994). Assessment of left ventricular function by the midwall fractional shortening/end-systolic stress relation in human hypertension. J. Am. Coll. Cardiol..

[17] Aurigemma G.P., Silver K.H., Priest M.A., Gaasch W.H. (1995). Geometric changes allow normal ejection fraction despite depressed myocardial shortening in hypertensive left ventricular hypertrophy. J. Am. Coll. Cardiol..

[18] Gaasch W.H., Battle W., Oboler A.A., Banas J.S., Levine H.J. (1972). Left ventricular stress and compliance in man. With special reference to normalized ventricular function curves. Circulation.

[19] Mirsky I., Pfeffer J.M., Pfeffer M.A., Braunwald E. (1983). The contractile state is the major determinant in the evolution of left ventricular dysfunction in spontaneously hypertensive rat. Circ. Res..

[20] Gaasch W.H., Zile M.R., Hoshino P.K., Apstein C.S., Blaustein A.S. (1989). Stress-shortening relations and myocardial blood flow in compensated and failing canine hearts with pressure-overload hypertrophy. Circulation.

[21] Schwarz F., Flameng W., Langebartels F., Sesto M., Walter P., Schlepper M. (1979). Impaired left ventricular function in chronic aortic valve disease: Survival and function after replacement by Björk-Shiley prosthesis. Circulation.

[22] Gerdts E., Rossebø A.B., Pedersen T.R., Cioffi G., Lønnebakken M.T., Cramariuc D., Rogge B.P., Devereux R.B. (2015). Relation of left ventricular mass to prognosis in initially asymptomatic mild to moderate aortic valve stenosis. Circ. Cardiovasc. Imaging.

[23] Mureddu G.F., Cioffi G., Stefenelli C., Boccanelli A., de Simone G. (2009). Compensatory or inappropriate left ventricular mass in different models of left ventricular pressure overload: Comparison between patients with aortic stenosis and arterial hypertension. J. Hypertens..

[24] Aurigemma G.P., Devereux R.B., De Simone G., Roman M.J., O’Grady M.J., Koren M., Alderman M., Laragh J. (2002). Myocardial function and geometry in hypertensive subjects with low levels of afterload. Am. Heart J..

[25] Palmieri V., Wachtell K., Gerdts E., Bella J.N., Papademetriou V., Tuxen C., Nieminen M.S., Dahlöf B., de Simone G., Devereux R.B. (2001). Left ventricular function and hemodynamic features of inappropriate left ventricular hypertrophy in patients with systemic hypertension: The LIFE study. Am. Heart J..

[26] De Simone G., Verdecchia P., Pede S., Gorini M., Maggioni A.P. (2002). Prognosis of inappropriate left ventricular mass in hypertension: The MAVI Study. Hypertension.

[27] Galderisi M., de Simone G., Cicala S., De Simone L., D’Errico A., Caso P., de Divitiis O. (2003). Coronary flow reserve in hypertensive patients with appropriate or inappropriate left ventricular mass. J. Hypertens..

[28] Palmieri V., Okin P.M., de Simone G., Bella J.N., Wachtell K., Gerdts E., Boman K., Nieminen M.S., Dahlöf B., Devereux R.B. (2007). Electrocardiographic characteristics and metabolic risk factors associated with inappropriately high left ventricular mass in patients with electrocardiographic left ventricular hypertrophy: The LIFE Study. J. Hypertens..

[29] De Simone G., Gottdiener J.S., Chinali M., Maurer M.S. (2008). Left ventricular mass predicts heart failure not related to previous myocardial infarction: The Cardiovascular Health Study. Eur. Heart J..

[30] Davies C., Zerebiec K., Rożanowska A., Czestkowska E., Długosz D., Chyrchel B., Surdacki A. (2018). Is left ventricular hypertrophy a friend or foe of patients with aortic stenosis?. Postepy Kardiol. Interwencyjnej.

[31] Agoston-Coldea L., Bheecarry K., Cionca C., Petra C., Strimbu L., Ober C., Lupu S., Fodor D., Mocan T. (2019). Incremental predictive value of longitudinal axis strain and late gadolinium enhancement using standard CMR imaging in patients with aortic stenosis. J. Clin. Med..

[32] Bang C.N., Greve A.M., Køber L., Rossebø A.B., Ray S., Boman K., Nienaber C.A., Devereux R.B., Wachtell K. (2014). Renin-angiotensin system inhibition is not associated with increased sudden cardiac death, cardiovascular mortality or all-cause mortality in patients with aortic stenosis. Int. J. Cardiol..

[33] Goh S.S., Sia C.H., Ngiam N.J., Tan B.Y., Lee P.S., Tay E.L., Kong W.K., Yeo T.C., Poh K.K. (2017). Effect of renin-angiotensin blockers on left ventricular remodeling in severe aortic stenosis. Am. J. Cardiol..

